# Everybody's Page

**Published:** 1892-05-14

**Authors:** 


### Everybody's Page.

The bite of a snake which is running away is said to be less dangerous than that of one which is aggressive.

A surgeon who was refused his fee after having stitched up a patient's wound discovered a simple remedy for the refusal by at once cutting the stitches and declining to do any more work for nothing.

According to Dr. Lauder Brunton, cold water is a valuable stimulant to many if not all people. Its action on the heart is more stimulating than brandy. His own experience is, that sipping half a wine glass of cold water will raise his pulse from 76 to over 100.

Electricity bids fair to do much for human nature when, if ever, it is completely understood and under control. Though at present it has not been found possible entirely to remove organic matter from water by its means, it has the power to reduce such matter appreciably, and now a Russian physician asserts that the pain of neuralgia, if superficial, can be relieved at once by throwing a beam of light from a bright arc-light upon the affected part.

Whilst in some quarters in this country doubts are being constantly thrown upon the benefits derivable from vaccination, and clamours are raised for its abolition as a compulsory prophylactic, in France, the Minister of the Interior, taking to heart the valuable results of past experience, to which some people seem wilfully blind, has introduced a law making vaccination obligatory during the first year of life, and re-vaccination in the course of the tenth and twenty-first year.

The editor of *The Gentlewoman* has asked his readers to reply to the question "Do Women desire to Vote for Members of Parliament?" by voting on the subject, and as soon as possible he will announce the total votes on either side. Mr. Gladstone has affirmed that until the "womanly mind" of the country declares itself unmistakably in favour of having the power to vote, that should not be enfranchised. As *The Gentlewoman* is not a political journal it will be enabled to get at the views of a large and representative and thinking body of women, non-political as well as political.

It is evidently hopeless to escape from microbes. Even the Dead Sea is not safe, and should be re-named, for it is stated by a Frenchman to be very much alive with microbes of gangrene and tetanus!

An interesting statement has been published by a French army doctor to the effect that the regularity of the marching step in the army has a most deleterious effect upon the health of even the strongest soldiers. The regularity of the step causes the indefinite repetition of a shock to the brain which does not occur in the ordinary irregular walk. As a preventive of this shock he has suggested the attachment of a rubber heel to all military boots, with which experiments are now being made in the French army to the undoubted relief of the soldiers.

It is alleged that on a certain road in the South of England there is a public-house where children are encouraged to drink by presenting a packet of sweets to the child who buys the most beer in the week. It is hard to believe such an allegation can have any foundation, but no doubt when licensing day comes round the magistrates will have something to say in the matter. Little wonder that so much misery and crime exist if people, who are not worthy of the name "human," can be found willing to make a living out of the destruction of the innocence of childhood.

Like most things in China, the practice of surgery differs considerably from that in vogue in less enlightened western countries. Bone-setting in the Celestial Empire is a complicated affair, and doubtless much more efficacious than European methods. In setting a fractured limb the surgeon does not attempt to bring the bones together, but merely wraps the limb in red clay, inserting some strips of bamboo into the clay. These strips are swathed in bandages, and in the outer bandage the head of a live chicken is placed. Here comes in the superior science of the Celestial. After the bandage has been secured the fowl is beheaded, and its blood is allowed to penetrate the fracture, for it nourishes the fractured limb, and is "heap good medicine."

